# Gut Microbiome Assembly and Its Consequences for Disease Susceptibility in Fish

**DOI:** 10.3390/ani16213500

**Published:** 2026-11-05

**Authors:** Gayatree Behera, Sabine E. Hammer, Astrid S. Holzer, Mona Saleh

**Affiliations:** 1 Department of Farm Animals and Veterinary Public Health, Division of Fish Health, University of Veterinary Medicine, 1210 Vienna, Austria; gayatree.behera@vetmeduni.ac.at (G.B.);; 2 Department of Biological Sciences and Pathobiology, Immunology, University of Veterinary Medicine, 1210 Vienna, Austria; sabine.hammer@vetmeduni.ac.at

**Keywords:** microbiomes, fish health, probiotics, gut microbiome, sustainable aquaculture

## Abstract

The assembly of the gut microbiome in fish is a dynamic and tightly regulated process influenced by host genetics, early-life environmental exposure, diet, and aquaculture practices. Increasing evidence indicates that deliberate manipulation of this assembly through probiotics, prebiotics, synbiotics, environmental microbiome management, and husbandry interventions can significantly alter host–microbe interactions and downstream health outcomes. This review synthesizes we have summarized the current understanding about the regulatory principles of gut microbiome assembly in fish and explored the impact of manipulating the microbiome assembly process. Emphasis will be given to the early-life microbial colonization process, which is an important time point for microbiota succession and plays an important role in the development of immunity and resistance against diseases. Aquaculture systems, like recirculating aquaculture system (RAS) and flow-through system, where there is variation in microbiome exposure and water quality, further influence the process of microbiome assembly and pathogen dynamics. Several manipulative techniques, such as live feed enrichment, the seeding of microbes, and the management of water microbiomes, have been successful in inducing the growth of beneficial microbes while suppressing opportunistic pathogens. However, the risk of microbiome dysbiosis, reduction in microbial resilience, and pathogen resistance due to microbiome manipulation cannot be overlooked. Understanding these trade-offs is essential for developing sustainable and effective strategies. By integrating insights from microbiology, immunology, and aquaculture science, this review carries out a systematic PRISMA analysis, highlights the consequences of microbiome manipulation on disease susceptibility, and provides a framework for designing microbiome-informed interventions to enhance fish health and productivity in modern aquaculture systems.

## Simple Summary

The fish gut microbiome plays an important role in digestion, nutrient absorption, immune function, growth, and disease resistance. Its composition is influenced by fish species, developmental stage, diet, water quality, environmental conditions, and aquaculture practices. Beneficial microorganisms help prevent pathogen colonization and maintain intestinal health, while microbial imbalance can increase disease susceptibility. Strategies such as probiotics, prebiotics, synbiotics, fecal microbiota transplantation, and synthetic microbial consortia may improve microbial balance and fish health. Modern sequencing and multi-omics technologies help researchers understand microbial communities and their functions. Further research is needed to develop safe, effective, and sustainable microbiome-based strategies for aquaculture.

## 1. Introduction

Aquaculture is one of the most promising solutions to the increasing demand for protein in the world, and its success depends mainly on ensuring proper conditions for aquaculture [1]. The gut microbiome of fish represents a key factor in the regulation of host physiology, affecting nutrition, immunization, and disease tolerance. The gut microbiome of fish is extremely diverse and dynamic and plays an important role in metabolism, health, and fitness [2,3]. In terrestrial vertebrates, fish are constantly exposed to environmental microbiomes, and therefore the assembly of the gut microbiome becomes heavily dependent on exogenous factors such as water quality, diet, and habitat conditions [4]. Consequently, microbial colonization occurs predominantly through horizontal transmission, particularly during early developmental stages, making the microbiome highly adaptable but also sensitive to environmental perturbations. Accumulating evidence demonstrates that gut microbiota play a pivotal role in host growth, development, and immune function [5]. The relationship between early-life microbiome establishment and later disease susceptibility is summarized in Figure 1. The fish intestine hosts a complex consortium of mutualistic, commensal, and opportunistic microorganisms [3,6], which contribute to host physiology through enzymatic digestion, vitamin biosynthesis, and maturation of gut-associated immune responses [7,8]. These microbial communities also prevent pathogen colonization via competitive exclusion and antimicrobial metabolite production [9,10]. Conversely, dysbiosis can compromise host defense mechanisms and increase susceptibility to disease [11]. Dietary interventions further influence microbial composition, affecting growth performance, feed utilization, and immune responses [12,13].

Recent advances in high-throughput sequencing technologies, particularly 16S rRNA gene sequencing, along with multi-omics approaches such as metagenomics, metabolomics, and proteomics, have significantly enhanced our understanding of fish gut microbial communities [14,15,16,17]. These approaches have enabled a transition from taxonomic profiling to functional characterization, revealing microbial contributions to host metabolism, immune modulation, and pathogen resistance [18,19]. The gut microbiome is therefore increasingly regarded as a functional “organ” contributing to host homeostasis [20,21]. The fish gut microbiome is typically dominated by Pseudomonadota, Bacillota, Bacteroidota, and Actinomycetota, although their relative abundance varies with species, diet, developmental stage, and environmental conditions [13,16,19]. Phyla such as Gemmatimonadota, Cyanobacteriota, and Verrucomicrobiota are also reported, with Bacillota and Pseudomonadota often being the most abundant [22]. Actinomycetota are particularly important due to their role in synthesizing antimicrobial secondary metabolites; their depletion may weaken intestinal barrier integrity and immune responses [23].

Beyond the host, environmental microbiomes, including those in water and sediments, serve as reservoirs of both beneficial and pathogenic microorganisms in aquaculture systems. The water microbiome reflects interactions among nutrient inputs, organic matter, and microbial metabolism, influencing key processes such as nitrification, denitrification, and organic matter degradation [24]. Aquatic microbiomes encompass bacteria, archaea, fungi, viruses, and microalgae inhabiting both environmental and host-associated niches [25,26]. Bacteria dominate these communities and exhibit diverse metabolic strategies depending on gastrointestinal microenvironments [3].

Microbial diversity is a key determinant of ecosystem stability in aquaculture systems, with higher diversity associated with increased resilience to stress and disease outbreaks [27,28]. Consequently, microbiome manipulation strategies such as probiotics, prebiotics, synbiotics, biofloc systems, dietary modulation, and microbial water conditioning have been widely studied [12,29] and are increasingly being explored as sustainable alternatives to antibiotics [30], and have gained attention as a strategy to enhance fish health and aquaculture productivity. Emerging strategies such as microbiome engineering, microbiota transplantation, and AI-driven predictive modeling are further advancing targeted microbiome interventions [31]. Additionally, gut microbiota represents a potential source of bioactive compounds, including antimicrobial peptides (AMPs) [32]. A balanced microbiome enhances host defense by inhibiting pathogen colonization, producing antimicrobial metabolites, and stimulating immune responses [3,33]. In contrast, dysbiosis triggered by environmental stressors, pollutants, or poor management practices can disrupt microbial homeostasis and promote disease outbreaks [34]. Environmental factors such as antibiotics, seasonal variation, and pollution further exacerbate microbial imbalance [35,36]. Microbiome composition undergoes significant changes across developmental stages, with early life representing a critical window for microbial colonization and immune maturation. Studies in *Salmo salar*, *Oreochromis niloticus*, and *Cyprinus carpio* demonstrate stage-specific microbial profiles [4,37]. Environmental exposure through water and feed plays a key role in shaping early microbiome assembly, particularly in aquaculture systems such as recirculating aquaculture systems [38].

Given the rapid expansion of aquaculture and increasing concerns regarding antimicrobial resistance, microbiome-based strategies offer promising solutions for sustainable disease management [39,40]. Therefore, this review aims to synthesize current knowledge on the manipulation of gut microbiome assembly and its consequences for disease susceptibility in fish (Figure 1). By integrating the findings from eligible primary studies, this review addresses the factors affecting the gut microbiota, such as early-life microbial exposure, nutritional influences, environmental and aquaculture practices, and how these changes are associated with host health and susceptibility, while also addressing microbiome-mediated benefits and emerging technologies for the comprehensive characterization and manipulation of fish gut microbiomes.

## 2. Material and Methods

### 2.1. Data Sources and Search Strategy

The Preferred Reporting Items for Systematic Reviews and Meta-Analysis (PRISMA) 2020 guidelines were used to guide reporting in this review [41]. Primary research articles were retrieved from Science Direct, Scopus, PubMed, and Web of Science databases. All articles were retrieved in full text from 1 January 2015 to 15 April 2026 to make sure the latest applications of high-throughput sequencing were used, as well as ensuring that omics techniques for studying host–microbiome interactions, fish health and environmental microbial communities were included. . Certain key pre-2015 publications were cited where appropriate to provide historical background, establish core concepts, and support mechanistic arguments. Database-specific search strategies were designed using a variety of keyword combinations, connected by Boolean operators (and, or). A set of search terms was used to screen databases: fish gut microbiome or gut microbiota, gut microbiome in disease welfare in fish, fish gut microbiome and early life, fish gut microbiome manipulation, and fish gut microbiome and omics were used for the screening process in all databases. Results were restricted to peer-reviewed articles in English. No truncation or wildcard operators or controlled vocabulary were used.

### 2.2. Study Selection and Data Extraction

Article entries were first parsed and filtered based on abstract and title. Studies that addressed one or more of the major themes relevant to the review, including fish gut microbiome, fish health, probiotics or other microbiome-related interventions, disease, and omics-based approaches, were retained. Entries that mentioned or used other animal subjects besides aquatic life were removed. Thus, the presence of all keywords—“gut”, “microbiome”, “disease”, and “omics”—was not a mandatory inclusion criterion; rather, these terms were used to identify the main areas of relevance during the initial literature search.

Articles were further filtered to remove any entries that did not focus on fish for the study. Entries were also excluded if there were no full texts available, or if non-English articles were included. Duplicate records were removed accordingly. A formal risk-of-bias assessment and grading of study quality were not performed. The included studies encompassed heterogenous research designs, objectives, microbiome characterization methods, and host-related outcomes, making a single appraisal framework unsuitable for consistent application across the entire evidence base. No quantitative synthesis was performed because of this methodological and outcome heterogeneity. Consequently, findings were synthesized narratively, and limitations associated with study design, sampling, analytical methods, and the interpretation of associations were considered when discussing the evidence. The methodological characteristics and limitations reported in individual studies were considered when interpreting their findings. Experimental evidence was considered when discussing the strength and limitations of available evidence. After pre-evaluation, articles were downloaded based on availability and included in the final synthesis.

## 3. Results

### 3.1. Literature Search for Gut Microbiome in Fish Studies

In total, 373 records were identified through the combined database search, including 18 records from PubMed, 57 from Scopus, 29 from Web of Science, and 269 from Science Direct. Following the removal of duplicate records (n = 42), 331 records remained for screening based on their titles and abstracts. Of these, 187 records were excluded as they did not meet the predefined screening criteria. Subsequently, 144 reports were sought for full-text retrieval, of which 15 reports could not be retrieved. A total of 129 full-text reports were therefore assessed for eligibility according to the predefined inclusion and exclusion criteria. Following full-text assessment, 21 reports were excluded, while 108 studies met the eligibility criteria and were included in the final review (Figure 2).

### 3.2. Study Characteristic

Studies of fish that investigated the gut or intestinal microbiota and associated findings with fish health or welfare were considered. This included studies that described factors that influenced gut microbiome composition or function. The factors that were of interest included developmental stage, diet, environmental and rearing conditions, aquaculture systems, and microbiome-targeted interventions, were also considered. For inclusion, studies utilized culture-independent methods for characterizing fish microbiomes. That is to say that all studies using 16S rRNA gene sequencing, as well as other metagenomic sequencing, meta-transcriptomic sequencing, etc., were considered for inclusion. Both experimental and observational studies were also included when sufficient information on fish characteristics, microbiome assessment, and health- or welfare-related outcomes were available. Studies with non-fish species, microbial communities outside the gut (without a corresponding gut component), or with outcomes not relevant to fish health and/or welfare were excluded from this systematic review. Abstracts presented at conferences, reviews, editorials, and opinion pieces, as well as studies lacking relevant primary data, were excluded. Review articles were used for background information and reference-list screening but were not included as primary studies in the final synthesis.

## 4. Host and Environmental Microbiomes

In addition to being associated with the host, environmental microbiomes such as those present in the water column and in sediments also act as reservoirs of both beneficial and pathogenic microorganisms in aquaculture systems. The water microbiome is dynamic and is affected by nutrient inputs, by the associated organic matter, and microbial metabolism, influencing processes like nitrification, denitrification, and organic matter degradation [24]. Aquatic microbiomes encompass the complex communities of microorganisms, including bacteria, archaea, fungi, viruses, and microalgae—that inhabit aquatic environments and host-associated niches such as fish gut, skin, gills, surrounding water, and sediments. The majority of these consist of bacteria, and they can be aerobic, facultatively anaerobic, or obligately anaerobic, depending on the specific microenvironment within the GI tract [3]. These microbial assemblages play pivotal roles in maintaining the health, nutrition, and disease resistance of aquatic organisms while also influencing ecosystem productivity and water quality [25,26]. Gut microbes are very crucial in maintaining the average health of the fish and preventing pathogens from flourishing [9,10]. In fish, the gut microbiome is the most studied compartment, comprising symbiotic microorganisms. However, there has been a surge in attention to understanding fish gut microbiomes in the last decade due to their ecological and economic importance [2,42,43]. It is dominated by phyla such as Pseudomonadota, Bacillota, Bacteroidota, and Actinomycetota, whose relative abundance can vary depending on diet, species, and environmental factors [13,19]. Other notable phyla include Gemmatimonadota, Cyanobacteriota, and Verrucomicrobiota. Bacillota is the most abundant phylum, followed by Pseudomonadota [22]. The phylum Actinomycetota is important given its role in synthesizing secondary antibiotic metabolites toward aggressive pathogens; its reduction potentially leads to the weakening of the intestinal barrier and an increased sensitivity to immune stimulus [23]. Sediment microbiomes, often dominated by Pseudomonadota, Chloroflexota, and Bacteroidota, contribute to nutrient recycling, pollutant degradation, and the stabilization of aquaculture pond ecosystems [44]. They aid in digestion, nutrient absorption, and immune regulation. The gut-associated lymphoid tissue (GALT) in fish is particularly rich in T cells, predominantly CD8^+^ T cells, that constitute nearly 50–70% of immune cells in the GALT of *Cyprinus carpio* [45]. Understanding the role of these microbiomes is critical in developing sustainable, antibiotic-free strategies for disease control [16,19]. The composition of fish microbiota varies with species, developmental stage, diet, and environment [3]. Beneficial bacterial taxa such as *Lactobacillus*, *Bacillus*, *Pseudomonas*, and *Aeromonas* (non-pathogenic strains) often dominate healthy fish intestines and contribute to digestion, nutrient absorption, and immune modulation [46]. Environmental factors, including water temperature, salinity, and stress, can significantly alter microbiome stability [47].

Comprehending the importance of the role of microbial diversity in aquaculture settings is key to formulating sustainable strategies for their management. The advent of high-throughput sequencing technologies and metagenomics has facilitated detailed analyses of microbial populations like never before. It has been reported that microbial diversity is crucial for the functional stability of systems. Aquaculture systems with higher microbial diversity are more resistant to environmental stresses and disease outbreaks compared to systems with lower diversity [27,28]. Moreover, the manipulation of the microbiome in probiotics, prebiotics, and biofloc systems is becoming increasingly important as it provides an environmentally sustainable approach to using antibiotics by promoting beneficial microbes and inhibiting pathogens [30]. It can be concluded that aquatic microbiomes form the basis of health, stability, and productivity of aquaculture. A deeper understanding of their structure, dynamics, and interactions offers transformative potential for advancing sustainable fish farming practices and mitigating emerging challenges in aquatic animal health and ecosystem management.

## 5. Gut Microbiome Dynamics Across Ontogenetic Life Stages in Fish

The fish gastrointestinal microbiome influences host physiology with respect to nutrition, immune system, disease resistance, and fish quality [2,3,48]. The composition and diversity of gastrointestinal microbial symbionts are influenced by various intrinsic and extrinsic factors including host age, host genetics, host diet, environmental factors and gastrointestinal niche specialization [37,38,49,50]. Among these, host developmental stages are a key determinant, particularly in teleosts, which undergo complex ontogenetic transitions [51,52]. However, the long-term influence of early-life microbiome assembly on adult gut structure remains relatively understudied [52]. Figure 3 shows the study conducted on the juveniles of *Schizopygopsis younghusbandi* to understand the host–microbiome–environment interactions in terms of phyla. Where the fungal communities were mainly comprising Ascomycota and Basidiomycota, while bacterial populations were predominantly made up of Pseudomonadota, Bacteroidota, and Bacillota. Algal communities were primarily represented by *Chlorophyta* and *Bacillariophyta*, whereas protozoan populations were mostly associated with *Ciliophora* and *Cercozoa*. The microbial communities found in water and fish guts exhibited significant differences, with only 10.3% of bacterial taxa being common between the two environments, indicating strong environmental filtration and habitat-specific assembly of microbial communities [53].

Early life stages (egg, larval, and fry) represent critical windows for microbial colonization, where host–microbe interactions are highly dynamic and sensitive to environmental perturbations [54]. During these stages, microbial communities undergo rapid succession driven by developmental processes and feeding transitions [55,56]. For example, studies on channel catfish have shown that eggs are initially dominated by genera such as *Vibrio* and *Flavobacterium*, which shift dramatically after hatching to taxa like *Opitutus*, followed by increased diversity during first feeding stages [57]. Similar ontogenetic microbial transitions have been documented in Atlantic salmon, zebrafish, and Atlantic cod, highlighting stage-specific microbial signatures [51,56,58].

Dietary shifts, particularly the onset of exogenous feeding, strongly influence gut microbiota composition, often overriding environmental microbial inputs [38,59]. This transition marks a key period where microbial communities shift toward taxa derived from feed sources rather than water [60]. Notably, the gut microbiome stabilizes as fish reach later developmental stages, with dominant taxa such as *Clostridium*, *Cetobacterium*, and *Turicibacter* forming a relatively stable core microbiota [57]. At higher taxonomic levels, phyla such as Pseudomonadota, Bacillota, Bacteroidota, and Actinomycetota dominate across multiple fish species and life stages [57,61,62]. However, their relative abundance varies significantly during development. For instance, Pseudomonadota often dominate early stages, while Bacillota and Fusobacteriota increase with maturation [63,64]. Studies in common carp further confirm that microbial composition changes dynamically during development, with Pseudomonadota and Bacillota showing fluctuating abundance patterns linked to host growth and physiological changes [65].

Importantly, early microbial colonizers play a pivotal role in shaping host immunity and long-term health outcomes by influencing epigenetic regulation and immune system maturation [66,67]. Disruptions in these early microbial interactions can lead to poor larval performance and increased disease susceptibility in aquaculture systems [55]. Therefore, understanding microbial succession across ontogeny is critical for developing microbiome-based interventions to enhance fish health, growth, and aquaculture sustainability [55,68].

## 6. Factors Affecting the Gut Microbiota

The gastrointestinal microbiome of fish is composed of a complex mixture of aerobic, facultative anaerobic, and obligate anaerobic microorganisms and can be influenced by a variety of intrinsic and extrinsic factors. Environmental factors (temperature, salinity), as well as farming practices, interact with host-related factors (age, genetics, and diet), playing critical roles in determining microbial diversity and stability. These groups of microbes are essential for a fish’s growth, metabolism, reproduction, behavior, and overall physiological performance. Disruptions in microbiome structure can therefore have cascading effects on host health and aquaculture productivity [19,69].

### 6.1. Feed-Driven Modulation of Gut Microbiome

Dietary manipulation can influence the fish gut microbial community composition, although the magnitude and direction of these changes depend on fish species, feed ingredients, inclusion levels, and feeding duration. Research exploring the inclusion of insect meal and the substitution of fishmeal has observed alterations in the structure of gut bacterial communities and the relative presence of certain taxa [70,71]. Conversely, some studies have noted more subtle effects, such as shifts in bacterial communities while fungal communities remain largely unchanged. These results indicate that dietary modifications can reshape specific microbial populations without ensuring a uniform change in overall beta diversity [72]. Figure 4 shows a study conducted on Nile tilapia to understand the effect of feed manipulation on the gut microbiome by supplying diets with 0.5% and 2% Chlorella vulgaris. The duration of feeding affected the composition of microbial communities, as samples collected on days 0 and 15 grouped together closely, while samples from day 30 formed a separate cluster [73].

#### 6.1.1. Probiotics

Dietary probiotics are among the most effective tools for manipulation of fish gut microbiota. The efficiency of probiotic microorganisms depends on specific traits of the used strains, such as spore-forming, adhesive properties, and production of antimicrobial compounds, e.g., bacteriocins, organic acids, and enzymes. The host benefits from these microbes in several ways: competitors to pathogenic microbes; modulators of host immune systems; and consumers of host nutrients that would otherwise be wasted by pathogens. *Bacillus subtilis*, for example, is a very effective pathogen inhibitor, largely due to its robustness and to the fact that it is a spore former, while *Lactobacillus plantarum* produces bacteriocins effective against *Vibrio* species [74,75].

Research indicates that *Rhizobium pusense* N7 has optimal denitrification efficiency and zebrafish survival at a concentration of 10^6^ CFU/mL [76]. Manuela Pillinger et al.’s investigation on the effects of single and multi-strain probiotics on the rainbow trout immune system found that multi-strain probiotics can have broader synergistic effects and immune-regulation effects than in pathogen invasion and inhibition, and MSP showed a stronger ability to trigger immune responses by activating the production of pro-inflammatory and anti-inflammatory cytokines in rainbow trout intestinal epithelial cells [77]. Ghasem Mohammadi et al. investigated the effects of single and mixed strains of probiotics on the growth of Nile tilapia cultured under different rearing conditions, and the results of the study revealed that mixed strains of probiotics could enhance the immune system of the fish by increasing the antioxidant system and improving gut health of the fish [78]. Similarly, Kong et al. explored the effects of the single and co-administration of *L. lactis* L19 and *Enterococcus faecalis* (*E. faecalis*) W24 in the diet on the health of *Channa argus*, and showed that *L. lactis* L19 was more effective than *E. faecalis* W24 and their mixtures in promoting these effects [79].

However, probiotic performance is often host-specific and influenced by environmental conditions, with variability in gastrointestinal survival (31–75% acid tolerance at pH 2–4) and colonization efficiency [80]. For example, *Bacillus subtilis* NTU-18 improves growth in *Anguilla japonica* glass eels but requires precise dosing for other species [81]. These limitations highlight the importance of precision microbiome engineering, including encapsulation technologies and host-adapted microbial consortia, to ensure consistent outcomes in aquaculture systems [82,83].

#### 6.1.2. Prebiotics

Prebiotics are soluble but non-digestible compounds that selectively stimulate the growth and/or activities of one or confined taxonomic groups of intestinal microorganisms, which can help to promote host health. Compounds such as chitosan oligosaccharides, polyphenols, and resistant starch have been shown to enrich beneficial taxa like *Lactobacillus* and *Bifidobacterium* while suppressing opportunistic pathogens such as *Vibrio* spp. [84]. These changes can improve gut barrier function, increase the body’s antioxidant capacity, and support the immune system. However, prebiotics are highly dose-dependent and can negatively affect the balance of microorganisms and thus have negative effects when used in excessive amounts, such as disrupting microbial equilibrium and reducing efficacy. Studies on striped catfish showed that the dietary insulin improved growth, feed efficiency, and several immune-related indicators, whereas in rainbow trout, which received a completely plant-based diet, inulin supplementation was associated with reduced growth and no significant changes in intestinal microbiota or plasma immune markers [85,86]. Other prebiotics such as mannan-oligosaccharides, β-glucans, and laminarin improved selected immune or antioxidant parameters in Nile tilapia and spotted seabass, although not significantly [87].

#### 6.1.3. Synbiotics

Synbiotics, a combination of probiotics and prebiotics, offer a synergistic approach to microbiome modulation by enhancing microbial survival and metabolic activity. In aquaculture, synbiotics have been shown to provide greater benefit than either single component and in aquaculture have been reported to improve growth, provide enhanced immune responses and protect against disease. The interaction between certain species of *Bacillus* and plant-derived prebiotics can be synergistic and reduce pathogenic *Vibrio* whilst promoting beneficial microbial species [88]. Prebiotics can support probiotic colonization and metabolize prebiotics to produce short chain fatty acids with immunomodulatory effects. Dietary supplementation with *B. velezensis* AAHM-BV2301 (1 × 10^8^ CFU/kg), chitosan (15 g/kg), and Silicon nanoparticles (SiNPs) has been reported to modulate the physiology, gut microbiota, and immune responses of Asian seabass, while enhancing resistance to *V. vulnificus* [89]. Advances in encapsulation technologies further enhance synbiotic stability and targeted delivery within the gastrointestinal tract, making them a promising tool for sustainable aquaculture [90,91].

#### 6.1.4. Fecal Microbiota Manipulation (Microbiome Transplantation)

Fecal microbiota transplantation (FMT) and synthetic microbial consortia (SynComs) represent emerging strategies for restoring or engineering gut microbial communities. FMT is the process of transferring fecal microbiota from a healthy donor into the gastrointestinal tract of a recipient to restore the composition and function of the recipient’s gut microbiota [92,93]. Synthetic microbial consortia are well characterized, scalable, and experimentally tractable. They provide various advantages over native microbial communities for predicting and identifying functionally relevant members and manipulating microbial communities [93,94]. In this review, we also consider approaches that involve the transfer of complete microbial communities or defined microbial consortia to set up stable communities of microorganisms that are beneficial to the host. The early-life microbial colonization of fish has a lasting effect on the gut microbiota of fish and thus can be manipulated for long-term effects. FMT has shown potential in restoring dysbiotic microbiomes and improving host resilience, particularly under intensive farming conditions [93]. A simplified synthetic microbial community enhances resistance of crucian carp (Carassius auratus) to Aeromonas hydrophila infection through host immune activation [95]. After fecal microbiota transplantation (FMT) treatment, fish larvae had significantly higher final body weight, weight gain rate, and specific growth rate. FMT treatment remarkably enhanced the activities of α-amylase and trypsin in the intestinal segment and the activity of lipase and trypsin in the pancreatic segment. Furthermore, FMT treatment significantly increased the activity of leucine-aminopeptidase on the brush border membrane and the mRNA expression of pcna and occludin compared with the control group. Similarly, the gut in larvae of the FMT group had significantly higher villus height and muscular thickness. Moreover, FMT treatment significantly improved the activity of lysozyme and decreased malondialdehyde concentration in visceral mass. FMT treatment increased intestinal microbial diversity and altered the intestinal microbiota profile of larvae [96]. Although epigenetic processes and cross-generational evidence, particularly from FMT, are still developing, early-life FMT modifies immune programming and may improve disease resistance [97,98].

Two Bacillus strains and Vibrio species decreased as the population of the community members increased in continuous multistrain coculture, exerting a more dominant influence on the growth performance of the groupers [99]. This would increase the abundance of potential probiotics and reduce the proportion of possible pathogens in the intestine of juvenile groupers. Lactis and Enterococcus faecium on the growth performance, digestive enzymes, and immunological response of the grouper Epinephelus coioides increased feed utilization and modified immune function [100].

Both methodologies need stringent biosafety regimes due to inherent dangers. FMT may aid in the horizontal gene transfer (HGT) of antibiotic resistance or virulence factors, especially if donors have dysbiotic or pro-inflammatory communities (e.g., carrageenan-induced microbiota worsening colitis) [101,102]. Recipient circumstances (e.g., food, water chemistry) do not match donor habitats, as demonstrated in interspecific transplants where nutritional alignment was crucial for stability [103,104]. Multi-omics-guided keystone taxon identification (such as Akkermansia for mucus integrity) reduces ecological disturbance during transplanting [105]. Insufficient knowledge of strain interactions in SynComs increases the possibility of community collapse or opportunistic taxonomic domination. Furthermore, environmental persistence of imported strains might affect native microbiomes or nutrient cycles [106]. Standardized donor screening, multi-omics-guided keystone taxon identification, and ecological risk assessments are all required for appropriate use. However, its performance is dependent on donor selection, host compatibility, and environmental stability, emphasizing the need for regulated and precision-based applications [107,108].

### 6.2. Environmental and Culture System Effects

The rearing environment plays a fundamental role in shaping the fish gut microbiome, particularly during early developmental stages. Fish larvae acquire microbes through water and feed, making the surrounding microbial community a primary determinant of gut colonization. Different aquaculture systems, such as biofloc systems (BFS), recirculating aquaculture systems (RASs), and flow-through systems (FTSs), expose fish to distinct microbial assemblages, resulting in variations in gut microbiota composition and diversity. NMDS ordination demonstrated shifts in gut and skin microbial community composition among experimental groups at the final sampling point (Figure 4). Significant differences in within-group dispersion relative to the control were also detected for both gut and skin microbiota.

The microbial populations present in RASs and FTSs were very similar, as was their behavior, throughout time. But in RASs, water is treated and reused, where biofilters provide surfaces for microbial attachment and growth, supporting the microbiome involved in water quality management, specifically for nitrification [109]. In contrast, in FTSs, there is continuous incoming water into the rearing system, creating a different input of microbial matter and dispersal. It has been reported that RASs show, theoretically, a greater capacity than FTSs to maintain and stabilize the microbial population in marine hatcheries [110], but differences between both systems in the present study, as statistical analysis supports, were irrelevant.

Biofloc systems, characterized by high microbial density and organic carbon supplementation, promote the development of beneficial microbial communities and enhance nutrient recycling. The microbial aggregates can also serve as a supplementary food source for cultured animals [111]. Fish reared in BFS often exhibit improved survival, growth, and microbial diversity compared to those in conventional systems. Experimental studies in Nile tilapia (*Oreochromis niloticus*) have shown that in situ biofloc production can increase gut microbial diversity and improve growth compared with biofloc-free controls [112]. In contrast, RASs and FTSs may limit microbial exposure, leading to less diverse microbiomes. Importantly, early-life microbial colonization influenced by these systems can have persistent effects, although host-driven selection tends to dominate microbiome composition as fish mature. Thus, manipulation of the rearing environment provides a powerful tool for microbiome management in aquaculture [54,55].

Biofloc systems, characterized by high microbial density and organic carbon supplementation, promote the development of beneficial microbial communities and enhance nutrient recycling. Fish reared in BFS often exhibit improved survival, growth, and microbial diversity compared to those in conventional systems. In contrast, RASs and FTSs may limit microbial exposure, leading to less diverse microbiomes. Importantly, early-life microbial colonization influenced by these systems can have persistent effects, although host-driven selection tends to dominate microbiome composition as fish mature. Thus, manipulation of the rearing environment provides a powerful tool for microbiome management in aquaculture [54,55].

### 6.3. Stress-Related Factors

Stress associated with aquaculture practices can affect the gut microbiota in a variety of ways. In the fish gut, environmental stressors such as handling, high stocking density, water temperature fluctuations, and other aspects of the fish environment can affect the gut via interaction with the Hypothalamus–Pituitary–Interrenal (HPI) axis. These influences can modulate between hormonal and immune aspects, and affect the balance between beneficial and opportunistic microbes, leading to shifts in dominant taxa. Early life stress, such as temperature shock during embryogenesis or adverse rearing conditions, can have long-lasting effects on the microbiota of fish. For example, stress exposure can shift gut microbial communities from dominance by beneficial groups such as *Bacilli* and *Lactobacilli* to opportunistic taxa within Gamma Pseudomonadota, including *Acinetobacter* and *Aeromonas*. These taxa are known for their resilience under stressful conditions and their ability to proliferate when microbial competition is reduced.

Furthermore, stress affects the gut–brain–microbiota axis, influencing neural, endocrine, and immune signaling pathways. In particular, the production of microbial metabolites, such as short-chain fatty acids, can affect neurotransmitter synthesis and stress response. On the other hand, stress hormones can affect the fish microbiota, but the precise mechanisms in fish remain under investigation; these bidirectional interactions highlight the importance of minimizing culture-related stress to maintain microbiome stability and optimize fish performance [113,114].

## 7. Molecular Methods for Assessing Microbial Communities in Aquaculture

Molecular methods have become fundamental tools for assessing microbial communities in aquaculture by enabling culture-independent identification, characterization, and functional evaluation of microorganisms across aquatic systems. For early molecular studies of microorganisms that did not require growth on agar, molecular techniques were employed for the detection and assessment of biodiversity of microorganisms by amplifying conserved genes such as the bacterial 16S rRNA genes and the eukaryotic 18S rRNA genes by PCR (Polymerase Chain Reaction) [115]. Molecular fingerprinting of environmental samples using denaturing gradient gel electrophoresis (DGGE) and temperature gradient gel electrophoresis (TGGE) allowed for comparison of microbial community structures, whereas fluorescence in situ hybridization (FISH) allowed for the direct visualization and localization of microorganisms [115]. Table 1 summarizes the evolution of molecular approaches applied in aquaculture microbiome research, including conventional PCR-based methods, fingerprinting techniques, high-throughput sequencing, and metagenomics. Information provided includes the study type, article title, molecular method(s) employed or discussed, their application in microbial community assessment, and corresponding references.

Next-generation sequencing (NGS) technologies have greatly facilitated the analysis of microbial communities by enabling high-throughput environmental DNA sequencing. Of these approaches, 16S rRNA gene amplicon sequencing is by far the most widely used method for taxonomic profiling and comparative analysis of microbial communities associated with aquaculture environments, fish, and shellfish microbiomes [119,131]. Functional prediction tools based on marker-gene data, such as PICRUSt, further extended the utility of amplicon sequencing by inferring microbial metabolic potential from taxonomic composition [130]. Recent methodological advances have incorporated long-read sequencing platforms, including PacBio sequencing, which provide improved taxonomic resolution and reduce amplification-related biases compared with conventional short-read technologies [116].

To overcome the functional limitations of marker-based approaches, shotgun metagenomics has become increasingly adopted in aquaculture microbiome studies because it enables simultaneous assessment of microbial composition and functional capacity through direct sequencing of total community DNA [129]. Metagenomics studies have enabled the identification of microbial pathways for nutrient cycling, metabolism, antimicrobial resistance, and host–microbe interactions in aquaculture systems [118,120,135]. Today, genome-resolved metagenomics also allows for the reconstruction of metagenome-assembled genomes (MAGs), which can be used for high-resolution microbial identification and ecological interpretation [117].

In addition to DNA-based approaches, microbial assessment increasingly incorporates methods targeting biological function and activity. Meta-transcriptomics enables analysis of actively expressed microbial genes, whereas proteomics, particularly matrix-assisted laser desorption ionization–time-of-flight mass spectrometry (MALDI-TOF MS), supports rapid microbial and pathogen identification in aquaculture systems [132]. Integrated multi-omics approaches, combining metagenomics with transcriptomics, metabolomics, and computational analyses, further provide comprehensive insights into microbial interactions and ecosystem function [133]. Moreover, combined molecular and metabolic monitoring approaches have demonstrated value for comprehensive evaluation of microbial quality and environmental conditions in aquaculture production systems [121]. Collectively, these advances illustrate the transition from traditional taxonomic characterization toward integrated, function-oriented microbial assessment frameworks that support sustainable aquaculture management.

### 7.1. Recent Applications of Omics Technologies in Microbiome Research in Aquaculture

Across the several studies, multi-omics strategies were implemented with varying degrees of integration and analytical complexity to investigate host–microbiome–environment interactions in aquaculture systems. Most of the recent studies, in particular those conducted as comprehensive systems-level analyses, have been performed by combining several types of omics data, such as transcriptomics, metabolomics, metagenomics, and/or SNP genotyping, as well as corresponding meta-transcriptomics and genome-resolved analyses to provide more complex biological processes and regulatory networks [136,137,138].

Previous studies on aquatic animal pathogens by Yang et al. (2025) and Thormar et al. (2024) were based on single-omics approaches, such as 16S rRNA gene-based profiling and genome-resolved metagenomics, which were supported by the functional prediction and reconstruction of meta-genome-assembled genomes [117,139]. The shotgun approach enables a more detailed analysis of the microbial community via the assessment of the taxonomic composition as well as the functional gene repertoire. Functional metagenomic studies in aquaculture, therefore, revealed the microbial functional potential in various aquaculture settings and host species and thereby identified pathways associated with nutrient turnover, polysaccharide utilization, microbial adaptation, and virulence [118,120,140].

Meta-transcriptomics provides an additional functional layer by capturing actively expressed genes. Cheng et al. (2025) established a mechanistic relationship between *Bacillus*-mediated lipid metabolism and enhanced antiviral immunity, showing that diglyceride production modulated immune signaling pathways [137].

Additionally, a number of studies focused on aspects of nutritional/feed-related research by applying different types of omics in an integrative manner, i.e., by using dual-omics designs, particularly transcriptomics coupled with metabolomics [141,142], enabling identification of metabolic reprogramming and molecular pathways associated with environmental stress responses and host immunity. The applications of omics in nutritional and feed-oriented studies [27,143,144] were further extended by the integration of transcriptomic and meta-transcriptomic analysis to examine the effects of dietary interventions on the activity of microbes, host metabolism, and performance. Recent studies, for example, demonstrated that rapid growth in hybrid fish is governed through an integrated gut–muscle–microbiome axis, where microbial metabolites, particularly AMP, were linked to activation of host growth-associated signaling pathways [145]. The studies of Toxqui-rodríguez et al. (2025) [146], and studies on nutrition and feed [27,143,144] used host transcriptomics and meta-transcriptomics to investigate the effects of different dietary interventions on activities of microbes, host metabolism, performance, rRNA profiling, and genome-resolved metagenomics, but strengthened biological interpretation through functional prediction and reconstruction of metagenome-assembled genomes [117,139].

Overall, the studies collectively present a substantial shift in methodology from simple descriptive taxonomic profiling towards a more integrated multi-omics approach that functionally characterizes host–microbiome–environment interactions. The emerging framework will support the development of precision aquaculture to promote health, productivity, and long-term sustainability. Table 2 summarizes representative studies highlighting research objectives, aquaculture species or production systems, microbiome or biological targets, omics methodologies employed, major findings, and corresponding references. Included studies demonstrate the application of multi-omics approaches for understanding microbial community structure, host–microbiome interactions, productivity, health management, and functional outcomes in aquatic organisms.

### 7.2. Emerging Technologies

Synthetic biology tools like CRISPR-based gene editing can be used to enhance the functionality of probiotics to remove nitrogen, inhibit pathogens, or even to produce vitamins [26]. In addition, AI-based microbiome modeling and computational ecology can enable aquaculture to achieve adaptive stability under changing environmental conditions [139]. The combination of engineered microbiomes with recirculating aquaculture systems (RASs) or biofloc technology offers great promise for the development of sustainable self-regulating systems that operate with little to no use of antibiotics and with low environmental impact. However, there are a number of issues that must be addressed, including gaining acceptance by regulators, concerns about ecological impact and stability, and ensuring consistent efficacy under field conditions.

Overall, these studies, taken together, define a substantial methodological shift from simple taxonomic profiling to functional multi-omics that allow for a holistic insight into host–microbiome–environment interactions. The emerging convergence of these approaches promises to support the development of precision aquaculture that promotes health, production, and long-term sustainability.

## 8. Gut Microbiome, Health, and Disease in Fish

Host-associated factors (genetics, immune status, developmental stage, and physiology) and environmental factors (water quality, diet, management practices, pollutants, and microbial exposure) collectively shape the gut microbiome composition and function (Figure 5). A balanced microbial community supports nutrient utilization, immune regulation, pathogen exclusion, and maintenance of intestinal homeostasis, whereas dysbiosis may contribute to impaired health and disease susceptibility.

### 8.1. Prophylactic Strategies

The innate immune system of fish is the first line of defense against pathogens and consists of a variety of effector cells, including macrophages, neutrophils, phagocytic cells, dendritic cells, and natural killer (NK) cells, along with a wide range of soluble immune factors that maintain mucosal immune homeostasis. Within this framework, the gut microbiome plays a central regulatory role in shaping immune equilibrium. Prophylactic modulation of the fish gut microbiome focuses on establishing and maintaining a stable, diverse, and functionally resilient microbial community to prevent disease onset. A well-balanced gut microbiota supports immune homeostasis, metabolic efficiency, epithelial integrity, and pathogen exclusion, thereby acting as the first line of defense against environmental and infectious challenges [151,152,153]. This stability is strongly influenced by external drivers such as diet, rearing environment, and husbandry practices, particularly during early developmental stages when microbial [54,154]. Dietary strategies using probiotics, prebiotics, and synbiotics are widely employed to proactively enhance beneficial taxa such as *Bacillus*, *Lactobacillus*, and *Cetobacterium*, which contribute to antimicrobial activity, competitive exclusion, and immune stimulation [75,90]. The use of probiotic formulas such as those containing species of the genus *Bacillus* can improve the survival of fish larvae as well as promote stability of gut microbiota in fish, such as Nile tilapia. Additionally, biofloc-based systems can promote the development of beneficial microbes within the system, enhancing the resistance of the disease [155,156]. These prophylactic interventions collectively aim to pre-empt dysbiosis by strengthening microbial network stability and reducing the likelihood of pathogen colonization under stress or environmental fluctuation.

### 8.2. Therapeutic Strategies

In aquatic organisms, host–pathogen interactions are increasingly characterized by disease-associated dysbiosis, that is, by decreased microbial diversity and an increased relative abundance of commensal, opportunistic, or pathogenic microorganisms. In Atlantic salmon suffering from amoebic gill disease (AGD), a marked reduction in bacterial diversity has been observed, accompanied by a strong enrichment of *Tenacibaculum dicentrarchi* within gill lesions, indicating a clear association between microbial imbalance and disease severity [157]. Similarly, in red abalone affected by withering syndrome, the digestive gland microbiome undergoes pronounced restructuring, where the relative dominance shifts from *Mycoplasma* to *“Candidatus Xenohaliotis californiensis”*. Importantly, several of the organisms found in healthy fish can cause disease in altered ratios of normal gut microbes [158]. Such findings reinforce the concept that dysbiosis, rather than single-pathogen invasion alone, underpins disease progression in aquatic hosts. The immune system plays a central role in maintaining microbial homeostasis through coordinated innate and adaptive mechanisms. In fish, intestinal macrophages regulate microbial stability, where their absence leads to dysbiosis, reduced *c1q* expression, and disruption of commensal communities, while immune restoration re-establishes microbial balance [159]. Adaptive immunity further contributes through T-cell-mediated tolerance mechanisms that prevent excessive immune activation against commensals [160]. Cytokine signaling is also critical, as demonstrated by interleukin-17A/F1 deficiency in Japanese medaka, which results in significant microbiome shifts, including enrichment of Verrucomicrobiota and Planctomycetota and dominance of *Plesiomonas shigelloides* [161]. Additionally, mucosal immunoglobulins such as IgT and IgD play conserved roles in microbial regulation by coating beneficial microbes, limiting pathobiont expansion, and shaping immune transcriptional responses, as shown in rainbow trout [162,163].

During disease states, microbial diversity consistently declines, often accompanied by an increase in opportunistic pathogens such as *Aeromonas*, *Vibrio*, and *Mycobacterium*, alongside reductions in beneficial taxa including Bacillota and Lactobacillales [164,165,166]. These dysbiotic shifts are frequently observed in bacterial and viral infections across fish species, highlighting conserved patterns of microbiome disruption. Therapeutic restoration strategies, including probiotics, postbiotics, and synbiotics, are therefore increasingly applied to re-establish microbial equilibrium, enhance immune responses, and suppress pathogen proliferation [91,167,168]. Probiotic taxa such as *Bacillus velezensis* and *Streptomyces* spp. have demonstrated strong potential in improving gut health, stimulating immune gene expression, and enhancing disease resistance in fish models. More advanced approaches, including fecal microbiota transplantation and synthetic microbial consortia, are emerging as next-generation microbiome engineering tools aimed at reconstructing severely disrupted microbial ecosystems and restoring functional stability [55,169]. Collectively, these strategies underscore a shift from pathogen-centric control to ecosystem-level microbiome management for improving fish health and aquaculture sustainability.

## 9. Future Perspectives

Despite substantial progress in elucidating fish gut microbiome dynamics, several critical knowledge gaps continue to limit the effective translation of microbiome research into aquaculture practice. A major priority is the development of predictive frameworks for microbiome assembly, particularly during early developmental stages. Early-life microbial colonization plays a decisive role in shaping host immune maturation, metabolic programming, and long-term disease resistance. Therefore, identifying keystone taxa and functional microbial traits that contribute to stable and beneficial community assembly remains essential.

Future research should increasingly adopt integrative multi-omics approaches, including metagenomics, meta-transcriptomics, metabolomics, and host transcriptomics, to move beyond compositional profiling toward mechanistic insights. Such integrative analyses will enable the identification of functional microbial pathways, host immune regulatory networks, and inter-microbial interactions underlying health and disease states. The integration of these datasets with machine learning, artificial intelligence, and systems biology frameworks will further facilitate predictive modeling of disease outbreaks and support precision aquaculture strategies.

Standardization of experimental design and analytical pipelines represents another critical requirement. Current variability in sampling strategies, DNA extraction methods, sequencing platforms, and bioinformatics workflows limits reproducibility and cross-study comparability. The establishment of standardized protocols, particularly for low-biomass samples such as water microbiomes, is essential for ensuring data reliability and enabling robust meta-analyses across species and production systems. Furthermore, the application of ecological theory frameworks may help shift microbiome research from descriptive characterization toward predictive and intervention-based models [170].

Environmental microbiomes, including water, biofilms, and live feed-associated microbial communities, should also be recognized as major drivers of gut microbiome assembly. In recirculating aquaculture systems (RASs), where microbial communities are continuously recycled, targeted manipulation of environmental microbiota offers a promising strategy to indirectly shape host-associated microbial ecosystems. This approach aligns with the emerging concept of microbial management and ecosystem engineering in aquaculture systems.

In parallel, the development of next-generation probiotics (NGPs) and precision microbiome engineering approaches represents a rapidly expanding frontier. Unlike conventional probiotics, NGPs are selected based on defined functional traits such as pathogen inhibition, immune modulation, and metabolic enhancement. Advances in synthetic biology and microbial consortium design may enable the development of tailored microbial assemblages optimized for specific host species and environmental conditions.

However, several scientific and regulatory challenges must be addressed prior to large-scale application. These include microbiome instability under dynamic environmental conditions, the risk of unintended dysbiosis, and the potential emergence of antimicrobial resistance associated with microbial manipulation strategies. Additionally, regulatory frameworks governing engineered or non-native microbial applications remain underdeveloped. Long-term, ecosystem-level studies are therefore required to evaluate the ecological safety, stability, and sustainability of microbiome-based interventions.

Finally, climate change and associated environmental stressors—including temperature fluctuations, hypoxia, pollution, antibiotic residues, and microplastics—are expected to exert profound effects on gut microbiome stability and host–pathogen interactions. A rise in mean temperature alters microbiome assembly and diversity, affecting intestinal barrier integrity, metabolism, and the immune system. Hypoxia, which may be exacerbated by temperature-driven reductions in oxygen solubility and eutrophication, can further compromise fish physiological performance and alter host–microbiome interactions. Integrating microbiome research with climate-resilient aquaculture strategies will be essential for ensuring future food security and sustainable aquatic production systems. Microplastics and pollutants have been associated with changes in oxidative stress and inflammation. Antibiotic residues may also exert selective pressure on the microbial communities, potentially favoring antibiotic-resistant bacteria and resistance genes. Future research should therefore investigate the combined and long-term effects of climate-related stressors on microbiome assembly and function across fish developmental stages.

Despite growing evidence on microbiome–host interactions, a fundamental gap remains in understanding how early-life microbial communities influence long-term gut microbiome stability and host performance. The persistence, functional resilience, and ecological succession of microbiota established during larval development are still poorly characterized, particularly under conditions of environmental transition, dietary shifts, and system transfer. Furthermore, the long-term implications of early microbial colonization on growth performance, immune competence, and disease resistance in cultured fish remain largely unresolved, highlighting a critical need for longitudinal and controlled experimental studies [88].

## 10. Conclusions

The gut microbiome’s assembly is a dynamic and central determinant of fish health, playing a crucial role in nutrient metabolism, immune regulation, growth performance, and disease susceptibility influenced by host developmental stage, diet, environmental conditions, and aquaculture practices. Accumulating evidence demonstrates that targeted manipulation of the gut microbiome through dietary interventions, probiotics, and environmental management can significantly enhance fish health and improve disease resistance. In addition, the molecular interactions between environmental microbiomes, fish, and pathogens remain largely elusive. There is a critical need to transition toward mechanistic and functionally driven microbiome research. The integration of multi-omics technologies with advanced computational tools, including systems biology and machine learning approaches, will be essential for establishing causal relationships between microbial communities and host physiological outcomes. Methodological heterogeneity across studies limits direct comparisons and generalizability, while reported associations do not necessarily establish causality. Standardized methods, adequate replication, and functional validations are needed to strengthen evidence linking gut microbiome assembly to fish health and disease susceptibility. Ultimately, microbiome-informed aquaculture represents a promising pathway toward sustainable and disease-resilient production systems and enables the development of targeted, environmentally sustainable strategies for improving fish health and productivity.

## Figures and Tables

**Figure 1:**
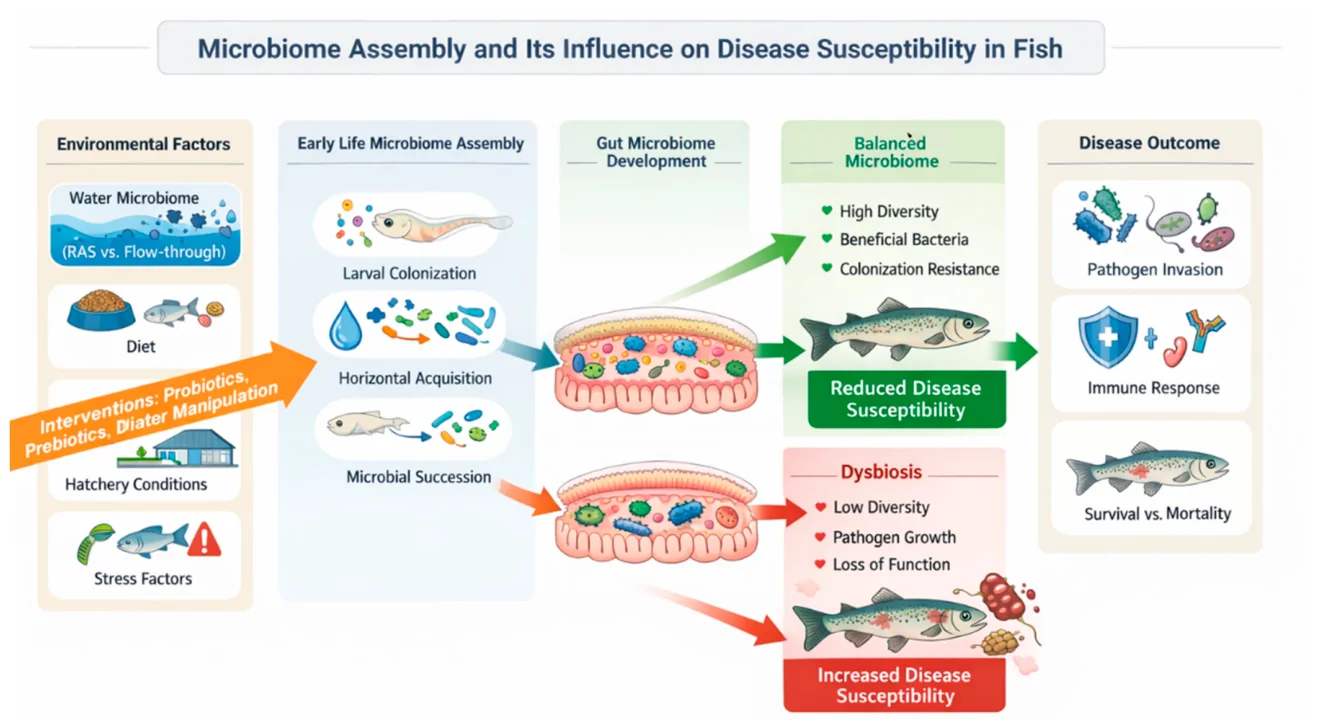
The role of microbiomes in early life and disease susceptibility. Created in Biorender.

**Figure 2:**
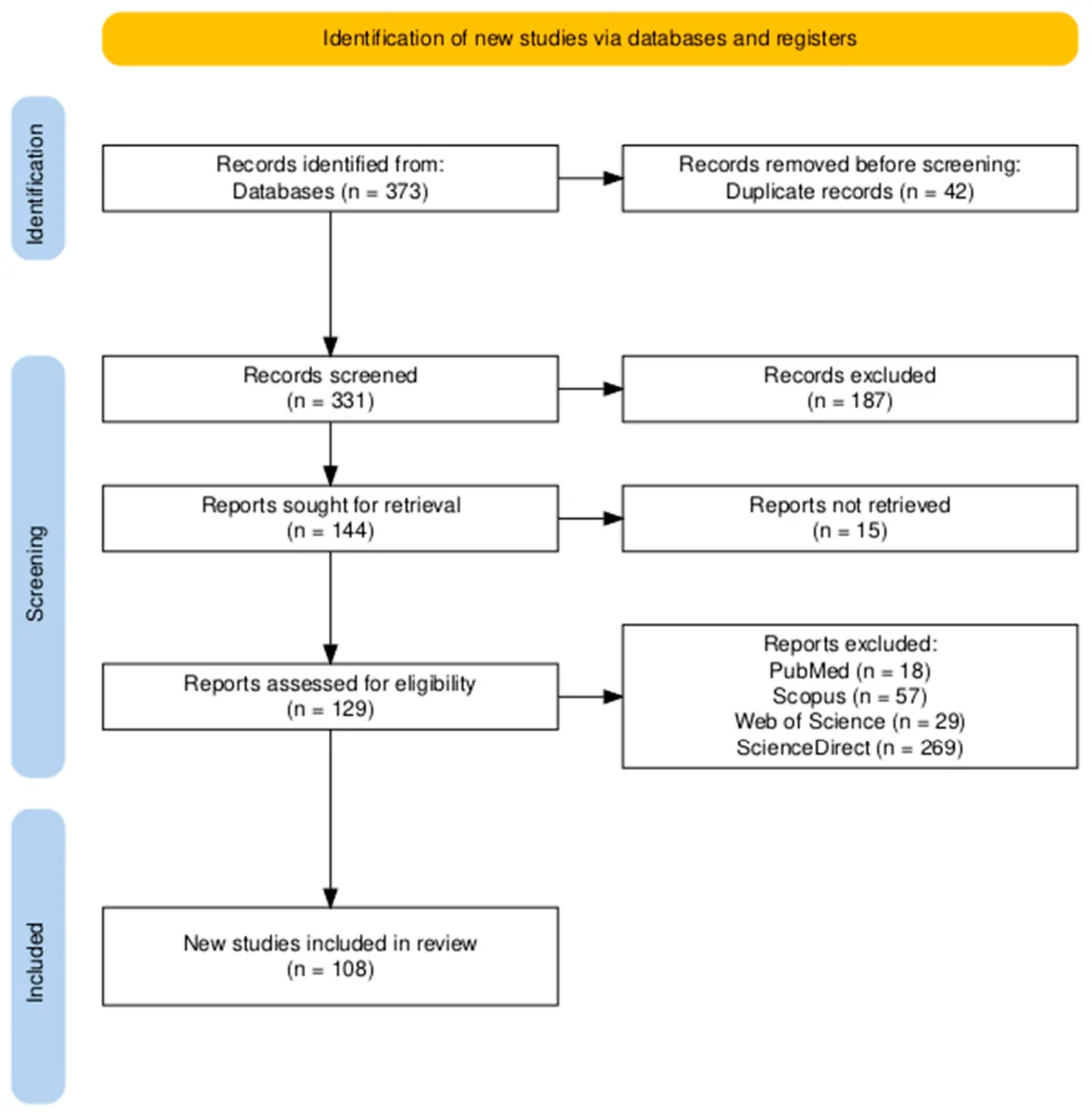
PRISMA flowchart of the literature search and inclusion process.

**Figure 3:**
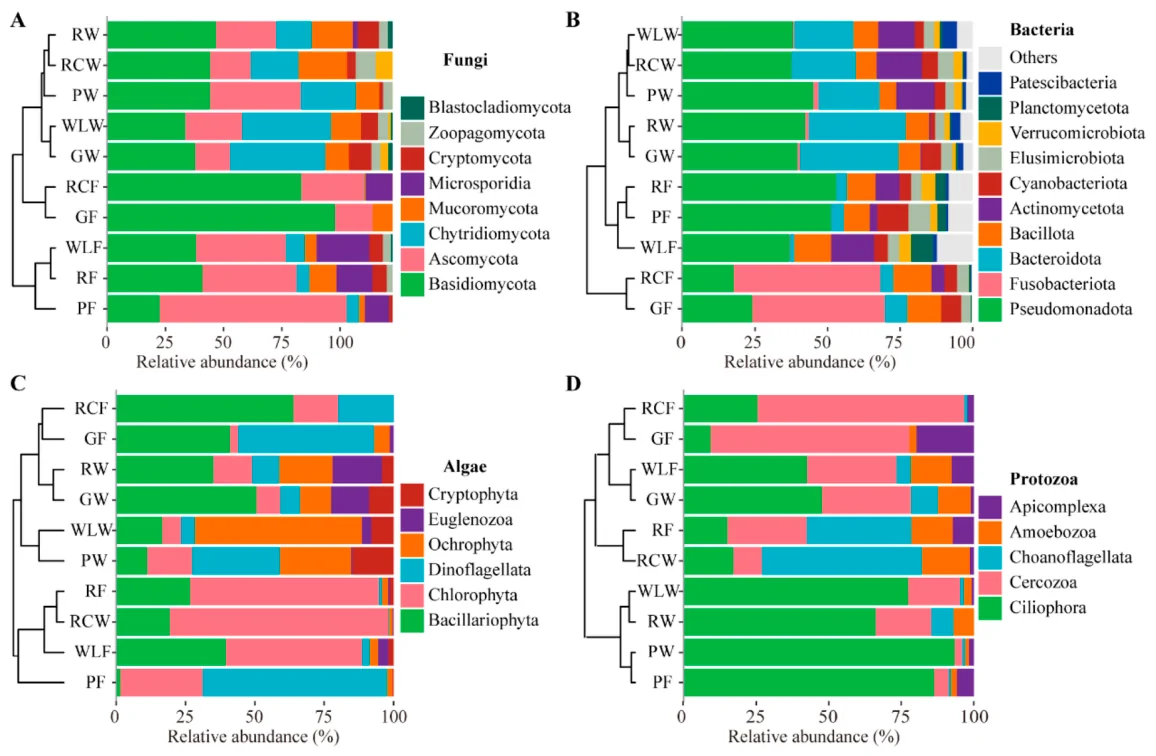
Microbial species composition in aquaculture water environments and the gut of juvenile *Schizopygopsis younghusbandi.* (**A**) Relative abundance of fungal phyla in water and gut microbiota. (**B**) Relative abundance of bacterial phyla in water and gut microbiota. (**C**) Relative abundance of algal phyla in water and gut microbiota. (**D**) Relative abundance of protozoan phyla in water and gut microbiota [53].

**Figure 4:**
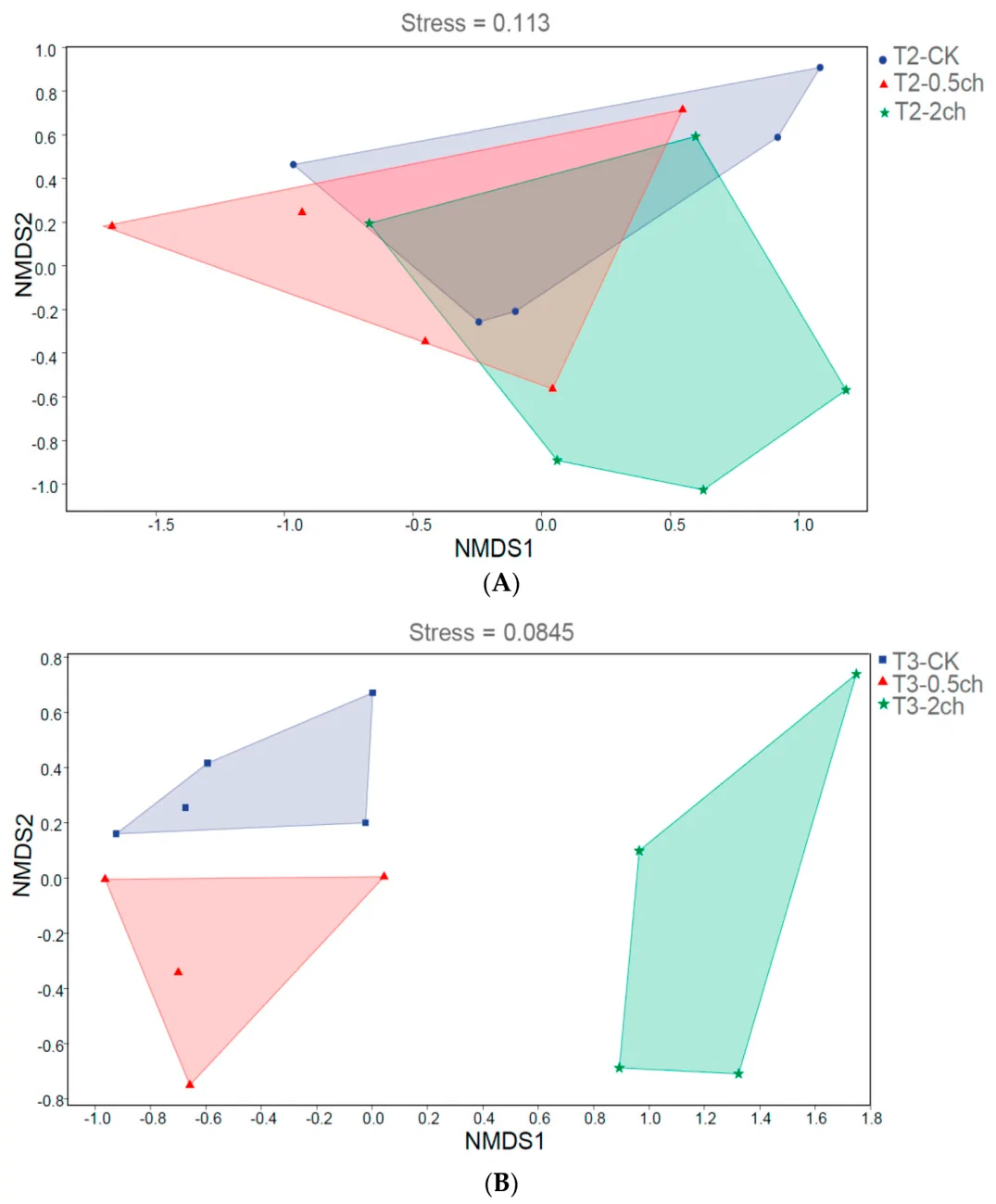
Nonmetric multidimensional scaling (NMDS) analysis of gut microbiota in Nile tilapia fed with different percentages of C. vulgaris. (**A**). NMDS analysis of the microbiota in Nile tilapia fed 0.5% and 2% C. vulgaris for 15 days. T2-CK, control group; T2-0.5ch, 0.5% C. vulgaris-treated group; T2-2ch, 2% C. vulgaris-treated group. (**B**). NMDS analysis of the microbiota in Nile tilapia fed 0.5% and 2% C. vulgaris for 30 days. T3-CK, control group; T3-0.5ch, 0.5% C. vulgaris-treated group; T3-2ch, 2% C. vulgaris-treated group [73].

**Figure 5:**
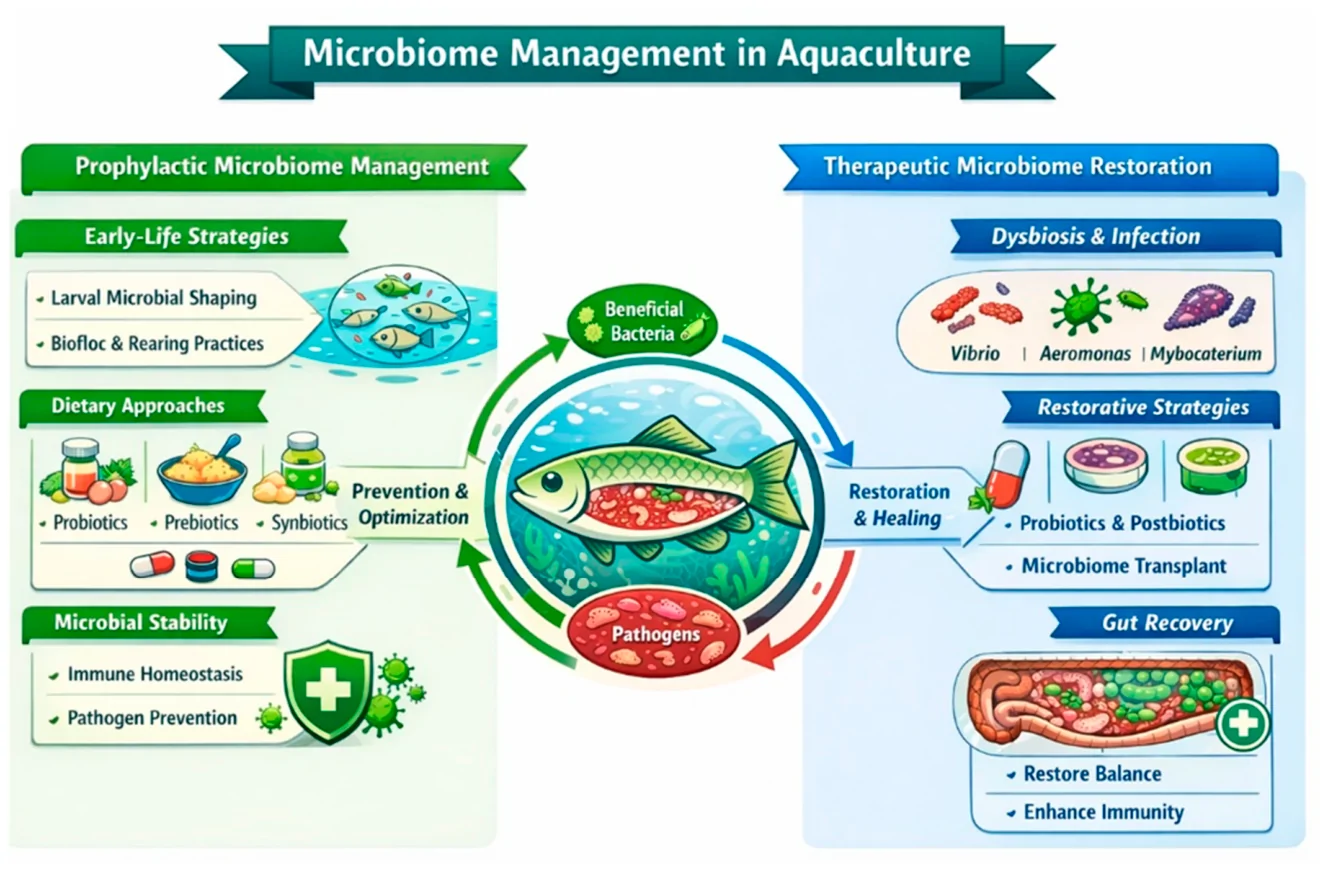
Schematic representation of gut microbiome–host–environment interactions in fish and the application of microbiome-based interventions for improving health and disease resilience in aquaculture systems. Created in Biorender.

**Table 1:** Molecular techniques used for the assessment of aquaculture-associated microbial communities.

Paper Type	Title	Molecular Method(s) Used/Discussed	Application in Microbial Community Assessment	Reference
Research	Metagenomics and metabarcoding experimental choices and their impact on microbial community characterization in freshwater recirculating aquaculture systems	Short-read 16S sequencing, PacBio long-read amplicon sequencing, shotgun metagenomics	Evaluation of the molecular methods for identifying microbial communities	[116]
Research	Sampling fish gut microbiota—a genome-resolved metagenomic approach	Genome-resolved metagenomics (MAGs), 16S metabarcoding	Optimization of microbial identification and retrieval from samples taken from fish intestine	[117]
Research	A metagenomic exploration of microbial distribution among pond sediment, shrimp tissue, and pond water	Shotgun metagenomics	Characterization of environmental and host-associated microbial communities	[118]
Research	Core species and interactions prominent in fish-associated microbiome dynamics	Quantitative 16S rRNA sequencing	Identification of microbial taxa and ecological interactions	[119]
Research	Comparative metagenomics of tropical reef fishes show conserved core gut functions across hosts and diets	Shotgun metagenomics	Functional and taxonomic assessment of fish gut microbiota	[120]
Research	An integrative, multiparametric approach for the comprehensive assessment of microbial quality and pollution in aquaculture systems	16S metabarcoding, metabolic profiling, microbial monitoring	Integrated assessment of microbial quality and community composition	[121]
Research	Deriving microbial community fingerprints from environmental samples using advanced molecular fingerprinting techniques	The application of molecular fingerprinting methods makes it possible to compare different microbial communities, identify community variations driven by diverse environmental changes, and yield a substantial amount of information on the microbial population.	Community composition of the organisms to understand the distribution, abundance, and structure of the microbiomes in environmental samples	[122]
Research	Molecular and culture-based assessment of the microbiome in a zebrafish (Danio rerio) housing system during set-up and equilibration	16S rRNA amplicon sequencing and culture using multiple agars and annotation of isolates via matrix-assisted laser desorption/ionization-time-of-flight (MALDI-TOF) mass spectrometry.	The bacterial community composition in the zebrafish housing systems and in the biofilters of the RAS when it has microbial equilibrium	[123]
Research	Comparison of Two 16S rRNA Primers (V3–V4 and V4–V5) for Studies of Arctic Microbial Communities	Targeting different hypervariable regions of the 16S rRNA gene (V3–V4 and V4–V5)	To understand the horizontal, vertical, and temporal turnover of microbial communities in the Arctic Ocean, which are poorly described	[124]
Research	Primer-free FISH probes from metagenomics/meta-ranscriptomics data permit the study of uncharacterized taxa in complex microbial communities	Attempts to bridge the gaps between metagenomics/meta-transcriptomics and high-resolution biomass imaging methods by developing new fluorescence in situ hybridization (FISH) probes—termed R-Probes—from shotgun reads that harbor hypervariable regions of the 16S rRNA gene	Techniques for studying individual species in complex microbial populations continue to be of utmost importance, especially for uncommon and/or new species that may have a significant role	[125]
Research	DNA metabarcoding across disciplines: sequencing our way to greater understanding across scales of biological organization	DNA metabarcoding describes the use of targeted DNA (i.e., amplicon) sequencing to identify community constituents from a complex sample containing genetic material from multiple organisms, such as water, soil, gut contents, microbiomes, or biofilms.	The SICB symposium brought together researchers using DNA metabarcoding techniques in order to demonstrate the applications of this technique for addressing questions related to ecology, evolution, and physiology and foster connections among researchers from various fields that are using similar approaches to facilitate optimization and standardization of metabarcoding methods and analyses.	[126]
Research paper	Molecular characterization of the intestinal microbiota of farmed Atlantic salmon (*Salmo salar* L.)	16S rDNA PCR, DGGE, sequencing	Characterization of intestinal bacterial communities	[127]
Research paper	PCR-TTGE Analysis of 16S rRNA from rainbow trout (*Oncorhynchus mykiss*) Gut microbiota reveals host-specific communities of active bacteria	PCR-TTGE; DNA- and RNA-based analyses	Examination of active bacterial communities and host-associated differences	[128]
Review	Molecular approaches to the assessment of biodiversity in aquatic microbial communities	PCR, 16S/18S rRNA markers, DGGE, TGGE, FISH, clone libraries	Identification and biodiversity assessment of aquatic microbial communities	[115]
Review	Metagenomics and other omics approaches to bacterial communities and antimicrobial resistance assessment in aquacultures	Shotgun metagenomics, meta-transcriptomics, proteomics, metabolomics, culturomics	Characterization of microbial diversity, function, and resistance genes in aquaculture	[129]
Review	Predictive functional profiles using metagenomic 16S rRNA data: a novel approach to understanding the microbial ecology of aquaculture systems	16S rRNA sequencing, predictive metagenomics (PICRUSt), bioinformatics	Functional analysis from microbial taxonomic data	[130]
Review	The complex microbiome in aquaponics: significance of the bacterial ecosystem	Next-generation sequencing (NGS), amplicon sequencing, microbiome profiling	Characterization of microbial ecosystems in aquaculture environments	[131]
Review	Use of proteomic-based MALDI-TOF mass spectra for identification of bacterial pathogens in aquaculture: a review	MALDI-TOF MS, proteomics	Rapid identification and classification of aquaculture-associated bacteria	[132]
Review	Microbiome and multi-omics insights into sustainable aquaculture: A triennial systematic review	16S sequencing, metagenomics, transcriptomics, metabolomics, multi-omics	Comprehensive assessment of microbial communities and functions	[133]
Review	Metagenomic profiling of fish-associated microbiota: ecological perspectives from freshwater to marine environment—a review	Metagenomics, microbiome profiling, sequencing-based ecology	Evaluation of fish-associated microbial communities	[134]

**Table 2:** Overview of omics-based studies investigating microbiome–host interactions and their applications in aquaculture systems.

Study	Objective	Species	Microbiome/Biological Focus	Omics Approach	Key Findings	Reference
Multi-omics reveals the molecular mechanisms of rapid growth in distant hybrid fish	Understand growth mechanisms through host–microbiota interactions	Hybrid fish	Gut–muscle–microbiota axis	16S + Metabolomics + Transcriptomics	AMP-mediated microbial signaling linked to mTOR/FoxO pathways	[136]
Multi-omics profiling reveals mechanisms of H_2_O_2_-induced effects on *Thamnaconus septentrionalis*	Explore oxidative stress responses	Greenfin horse-faced filefish	Gut microbiota	Transcriptomics+ Metabolomics+ Metagenomics	Oxidative stress altered immunity and microbial balance	[142]
Sampling fish gut microbiota—a genome-resolved metagenomic approach	Compare microbiome sampling strategies	Zebrafish	Gut microbiome	Genome-resolved Metagenomics + 16S	Feces yielded highest-quality MAGs	[117]
Transcriptomics and gut microbiome analysis of *Bidens pilosa* feed additive	Examine feed-mediated microbial regulation	Tilapia	Gut microbiome	Transcriptomics + 16S	Improved growth and metabolic regulation	[27]
Aquaculture ecosystem microbiome at the water–fish interface	Evaluate insect meal effects	Rainbow trout	Water–skin–gut microbiomes	16S sequencing	Reduced potential pathogens	[143]
Core species and interactions prominent in fish-associated microbiome dynamics	Identify microbial networks affecting fish performance	Japanese eel	Water-associated microbiome	Quantitative 16S	*Cetobacterium* associated with improved health	[119]
Deciphering the gut microbiome of grass carp through multi-omics	Construct functional gut gene catalog	Grass carp	Gut microbiome	Metagenomics + Transcriptomics	Defined microbial functional groups	[140]
Multi-omics analysis reveals *Bacillus* spp. enhance antiviral immunity	Understand probiotic-mediated antiviral mechanisms	Largemouth bass	Gut mucosal microbiome	16S + Transcriptomics + Metabolomics	Diglyceride-mediated activation of immune pathways	[137]
Multi-omics dataset of individual variations in growth performance	Explore growth variability	Large yellow croaker	Gut microbiome	Metagenomics + Transcriptomics + Metabolomics	Built microbiota–gene–metabolite networks	[147]
High-resolution gut microbiome characterization for sustainable aquaculture	Evaluate microbiota-directed feed	Atlantic salmon	Gut microbiome	Metagenomics + Transcriptomics + Meta-transcriptomics	Identified endogenous microbial targets	[144]
Interactions between gilthead seabream intestinal transcriptome and microbiota upon *Enteromyxum leei* infection	Explore host–microbiota–parasite interactions	Gilthead seabream	Intestinal microbiota	Host RNA-seq + 16S + Meta-transcriptomics	Identified microbial and transcriptomic biomarkers	[146]
Comparative metagenomics of tropical reef fishes	Assess dietary effects	Reef fishes	Gut microbiome	Metagenomics	Conserved gut functions across diets	[120]
Multi-omics integrative analysis reveals muscle adaptive changes underwater flow stress	Investigate physiological adaptation under recirculating aquaculture	Largemouth bass	Muscle metabolism	Transcriptomics + Metabolomics	Revealed calcium and Apelin signaling responses to flow stress	[148]
Multi-omics reveal the effect of dietary plant protein sources on the microbiota–gut–digestive gland axis	Assess alternative feed impacts	Abalone (*Haliotis dicus hannai*)	Gut–digestive gland axis	Multi-omics integration	Plant protein sources reshaped microbiota and host metabolism	[149]
Decoding the gut–microbiota–muscle nexus in rice–fish co-cultured common carp	Examine mechanisms for improving flesh quality	Common carp	Gut microbiota + muscle	Environmental microbiomes + Transcriptomics + Metabolomics	Identified microbiota-mediated activation of mTOR signaling	[150]

## Data Availability

Data sharing is not applicable to this article, as no new data were created or analyzed in this study.
